# Applications of the Wei-Lachin Multivariate One-Sided Test for Multiple Outcomes on Possibly Different Scales

**DOI:** 10.1371/journal.pone.0108784

**Published:** 2014-10-17

**Authors:** John M. Lachin

**Affiliations:** The Biostatistics Center, The George Washington University, Rockville, Maryland, United States of America; Cardiff University, United Kingdom

## Abstract

Many studies aim to assess whether a therapy has a beneficial effect on multiple outcomes simultaneously relative to a control. Often the joint null hypothesis of no difference for the set of outcomes is tested using separate tests with a correction for multiple tests, or using a multivariate *T* ^2^-like MANOVA or global test. However, a more powerful test in this case is a multivariate one-sided or one-directional test directed at detecting a simultaneous beneficial treatment effect on each outcome, though not necessarily of the same magnitude. The Wei-Lachin test is a simple 1 *df* test obtained from a simple sum of the component statistics that was originally described in the context of a multivariate rank analysis. Under mild conditions this test provides a maximin efficient test of the null hypothesis of no difference between treatment groups for all outcomes versus the alternative hypothesis that the experimental treatment is better than control for some or all of the component outcomes, and not worse for any. Herein applications are described to a simultaneous test for multiple differences in means, proportions or life-times, and combinations thereof, all on potentially different scales. The evaluation of sample size and power for such analyses is also described. For a test of means of two outcomes with a common unit variance and correlation 0.5, the sample size needed to provide 90% power for two separate one-sided tests at the 0.025 level is 64% greater than that needed for the single Wei-Lachin multivariate one-directional test at the 0.05 level. Thus, a Wei-Lachin test with these operating characteristics is 39% more efficient than two separate tests. Likewise, compared to a *T* ^2^-like omnibus test on 2 *df*, the Wei-Lachin test is 32% more efficient. An example is provided in which the Wei-Lachin test of multiple components has superior power to a test of a composite outcome.

## Introduction

In many studies an objective is to assess whether an experimental therapy (*E*) versus control (*C*) has beneficial effects on multiple component outcomes. This is becoming increasingly common in the evaluation of the comparative effectiveness of therapies. For example, the NIDDK-funded “**G**lycemia **R**eduction **A**pproaches in **D**iabetes: A Comparative **E**ffectiveness” (GRADE) Study will compare four agents commonly used to control glucose levels in type 2 (adult) diabetes [1], clinicaltrials.gov NCT01794143. The primary objective is to evaluate the durability of glucose control over 3–6 years of treatment, the primary outcome being the time to a confirmed rise of HbA1c (a measure of average glucose levels) ≥7% (the therapeutic target being a value <7%) using a logrank test. A secondary outcome is to compare each pair of treatments with respect to multiple components of effectiveness, specifically whether one treatment is superior to the other with respect to durability of control (event-times), absence of hypoglycemia over 3 years of treatment (proportions), and a lower mean body weight at 3 years. Herein we describe how such a test could be conducted and evaluate the power of the test or the required sample size.

For illustration, throughout we consider the case of two outcomes, say *A* and *B*, although all the procedures herein generalize to ≥2 outcomes. We wish to test the null hypothesis *H*_0_: (*A_E_*≡*A_C_*)∩(*B_E_*≡*B_C_*) that the experimental therapy is equivalent to control for both outcomes versus the alternative *H*_1_: 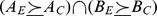 with at least one strict superiority, where “≡” means equality for an outcome and where “
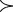
” means superiority. The test against such an alternative is called a multivariate one-directional (or one-sided) test.

Wei and Lachin [2] proposed a simple 1 *df* test for such a hypothesis that was described as a test against an ordered alternative, or a test of stochastic ordering. The test was later studied by Lachin [3] and Frick [4], [5]. Herein the application of this test to multiple outcomes is described for a test of means, a test of proportions, a test of event times and a test with mixed components such as where one outcome is quantitative (using means) and another qualitative (using proportions). For each application, equations are also derived for evaluation of sample size and power of the test. Multiple model-based tests are also described. For an analysis of multiple mean differences we show that the Wei-Lachin test is more powerful than an analysis based on either separate tests for each outcome, multiplicity adjusted, or a multivariate *T* ^2^-like omnibus test. An example from a major clinical trial is presented.

Many other tests have also been proposed, principally in the setting of tests for differences in means. These are reviewed in the discussion section.

### Wei-Lachin Multivariate One-Directional Test and Its Power

Three versions of the Wei-Lachin test are described. The first employs the measurements using the original scale of measurement. This test, however, is not invariant to scale transformations of the individual components. Two scale invariant tests are also described, one based on standardized values and another based on scale-independent *Z*-tests.

### Scale-Based Test For Multiple Outcomes

Let *X_ij_* designate the *jth* outcome variable in the *ith* group with expectation *E*(*X_ij_*) = *μ_ij_*, *i* = *E*, *C*; *j* = *a*, *b*. The subscripts *a*, *b* are used through out to refer to the two outcomes. The *j*th outcome could be a quantitative measure or a binary variable (among others). Assume that a more favorable outcome is represented by a decreasing expectation for *X*. Let
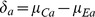
(1)

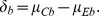



A positive value for each represents a beneficial effect of the experimental therapy over control for each outcome, and a negative value represents lack of benefit. The null and alternative hypotheses of interest are
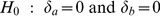
(2)

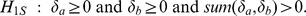



Thus, *H*_1S_ designates that the experimental therapy is at least as effective as control for both outcomes and is superior to control for either or both outcomes. This is called the multivariate one-directional hypothesis.

In the context of an analysis of repeated measures, or multivariate observations, Wei and Lachin [2] described a multivariate one-directional test, what they termed a test of stochastic ordering, i.e. a test of the null hypothesis that is directed towards an alternative hypothesis of the form *H*_1S_ in (2). Lachin [3], [6] contrasts this test with other tests, such as the omnibus test.

Consider group-specific estimates 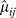 with expectation *μ_ij_*. Let 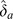 and 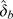 designate the estimates of the difference between the groups for each outcome as defined in (1), and 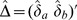, where “_′_” designates the transpose. With large samples
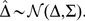
(3)with expectation 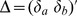 and with a covariance matrix 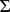 that is consistently estimable with elements
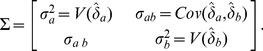
(4)


The Wei-Lachin test is then provided by
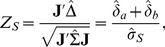
(5)

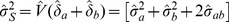
using consistent estimates of the variances and covariance, where 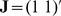. Asymptotically 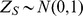 under *H*_0_ from Slutsky's theorem. The test rejects *H*_0_ in favor of *H*_1S_ when 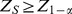 at level *α* one-sided. The above generalizes to *K*>2 outcomes. Note that the test can also be obtained from the unweighted average of the group differences relative to its standard error that provides a convenient average measure of the group differences when all outcomes are measured on the same scale.

Specific applications include a large sample test of means [3] or proportions [7], a generalized linear regression model using quasi likelihoods with a covariance matrix estimated using the information sandwich, i.e. GEE [8]; or a normal errors model for the analysis of repeated measures [9]; or a proportional hazards model using the information sandwich [10]; or these estimates can be based on a distribution-free estimate such as the Mann-Whitney difference that provides a Wilcoxon test [3], [11] with the Wei-Lachin [2] estimate of the covariance matrix. These and other methods allow for some observations for some outcomes in some subjects to be missing either completely at random or at random (conditionally).

Although often termed a multivariate one-directional (one-sided) test, it is possible to conduct a two-sided one-directional test that either *E* is superior to *C* for all components, or *C* is superior to *E*. In that case, the Wei-Lachin 1 *df* test statistic is referred to the two-sided critical value rather than the one-sided value. Herein we describe the one-sided test.

If beneficial values of *X_a_* are lower, but those for *X_b_* are higher, such as for a test of LDL and HDL, respectively, then the test would be constructed using the negative of the values for *X_b_* such that 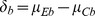. If higher values of both measures demonstrate benefit for the treatment, then both 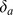 and 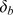 can be defined as the difference of treated minus control.

This test would be appropriate when all of the outcome measurements were on the same scale; for example, as for a test of a beneficial effect on both systolic and diastolic blood pressure (both mm Hg), or a test of a beneficial effect on both LDL and HDL (both mg/dl). Other variations described below would be appropriate for outcomes with different variances, or measures on different scales or mixtures of different types of measures, such as *A* being a quantitative variable and *B* being a binary variable.

An alternative approach commonly applied to test the superiority of an experimental therapy is to base the inference on the two separate one-sided tests. These tests would require a correction for multiple tests such as using the Holm [12] improved Bonferroni procedure which requires that the minimum of the two *p*-values be ≤0.025 (one-sided) and the other ≤0.05 in order to declare significance at the 0.05 level for the two tests. The corresponding alternative hypothesis is
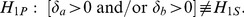
(6)


However, the alternative *H*_1*P*_ includes the case where the experimental therapy is beneficial for one outcome but harmful for the other, such as where 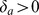 and 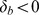 or vice versa.

Yet another possible test would be the omnibus test using a *T* ^2^-like test of the null hypothesis *H*_0_ versus
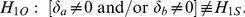
(7)that is provided by
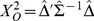
(8)which is asymptotically distributed as chi-square on 2 (or more generally *K*) *df*. This is likewise inappropriate because the alternative includes cases where the experimental therapy is worse than control for either or both outcomes.

### Maximin Efficiency of the Wei-Lachin Test

For the case of two measures as herein, the restricted alternative multivariate one-dimensional hypothesis *H*_1S_ in (2) corresponds to all points in the positive orthant of the two-dimensional parameter space for 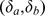. Since the test is a sum of the two estimates, the rejection region is defined by the line of values 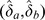 satisfying 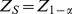 that simply connects the points 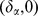 and 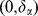 where 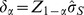. Thus the rejection region principally includes an area of the positive orthant away from the origin, but also includes elements of the sample space where either 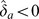 or 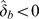, but not both. With large sample sizes, the probability of such points is negligible for true values 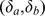 away from zero, i.e towards the central projection (the 45° line) of the positive orthant. Lachin [6] provides figures to illustrate these relationships.

For a given pair of values 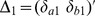 specifying a point in the positive orthant 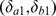, it is readily shown [13] that the optimal likelihood ratio test of *H*_0_: 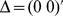 versus the point alternative 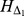: 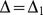 based on (3) is
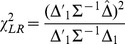
(9)where 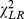 is distributed as chi-square on 1 *df* under *H*_0_. Note that 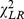 is based on a weighted sum of the estimated differences 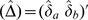. Thus, for a given 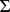, every point 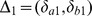 that defines a unique alternative hypothesis value in the two dimensional parameter space entails a different optimal linear combination of the observed 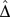. Further, the same weights are optimal for any alternative hypothesis defined by points proportional to 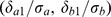 with the same correlation, such as the point 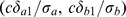 for any *c*>0. This implies that the same weights would be optimal for all points in the parameter space falling on the vector projection defined by the specified 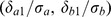. Thus, there are an infinite number of alternative hypotheses corresponding to all possible projections in the positive orthant, each with a different optimal test.

Unfortunately it is not known which projection is optimal since the actual parameter values 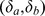 are unknown. However, Frick [4], [5] showed that the Wei-Lachin test is maximin efficient with respect to whichever weighted test is in fact optimal under the condition that 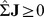. That is, among the family of linear combinations of the estimates, the Wei-Lachin test minimizes the loss in efficiency (power) relative to the unknown optimal linear combination when this condition applies, in which case it is the optimal robust linear test of *H*_0S_ versus *H*_1S_. For two or more measures with positive correlations, as would be the case under the alternative hypothesis, Frick's condition 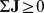 is satisfied.

When this simple condition does not apply, Frick [4] shows that a simple weighted test is provided by
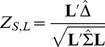
(10)that is also maximin efficient where **L** satisfies the restriction 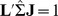. For a given 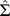, the vector **L** is obtained as 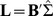 where **B** is the quadratic program solution to 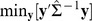 under the constraints that 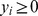 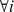 and 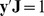. This test will principally be required in cases where the null hypothesis applies, or the treatment is inferior for some of the component outcome measures. A SAS program for this computation is available from the author (see Discussion).

### Scale-based Test for Multiple Means

To illustrate the construction of the Wei-Lachin test, consider a large sample test for a difference between groups in the means of two outcomes where it is assumed that 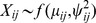 with some distribution *f* where 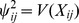 is the variance of the observations for the *j*th outcome in the *i*th group, or the residual variance after adjusting for other covariates, and 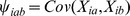, *i* = *E*, *C*; *j* = *a*, *b*. To simplify, assume that there is a common covariance matrix in the two groups (homoscedasticity) with correlation 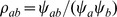. Then asymptotically
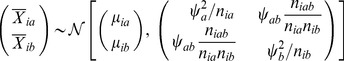
(11)where (*n_ia_*, *n_ib_*, *n_iab_*) are the numbers in the *i*th group with observed values for outcome *A* and *B* separately and jointly, *i* = *E*, *C* [3].

Then 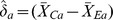 and 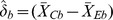 and 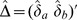 is asymptotically distributed as in (3) with covariance matrix
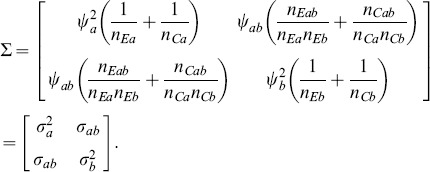
(12)where the variances 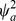, 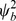 and covariance 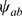 can be estimated directly from the available observations [3] under the homoscedasticity assumption. The estimated variance of the sum of mean differences is
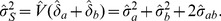
(13)


These then provide the test statistic *Z_S_* in (5), or *Z_S_*_,*L*_ in (10) if Frick's condition is not satisfied.

### Standardized Score Test for Multiple Means

For an analysis of the means of quantitative variables, the Wei-Lachin test *Z_S_* is not invariant to a change of scale for either of the two measures. In cases where there is a mixture of quantitative variables with different dispersions or units, such as LDL measured in mg/dl and systolic blood pressure measured in mm Hg, it is more meaningful to compute a scale-invariant test using the average of the corresponding standardized differences. This might also be preferred when the variances of the measures differ substantially, even though measured on the same scale.

Let *Y_ij_* denote the standardized value 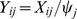 with 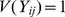. Then the standardized difference between groups for the *j*th outcome is
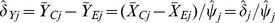
(14)where 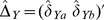 is asymptotically normally distributed with expectation 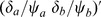 and covariance matrix
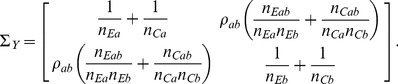
(15)


The resulting standardized Wei-Lachin test is then provided by
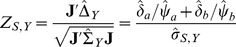
(16)where
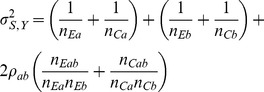
(17)that is consistently estimated from the estimate of the correlation 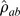. When the variances of the outcomes are equal 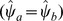, then 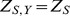. With equal sample sizes and no missing values, 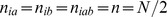, 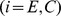, then
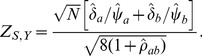
(18)


As above, with positive correlations, Frick's condition 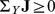 is satisfied. If not, then the weighted test is provided by 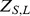 using 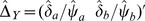 and 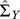 in (10).

### *Z*-Based Test

In some cases, it may be desired to conduct a test with mixtures of quantitative and qualitative outcomes (or other types), e.g. combining tests for means, proportions and/or life-times. In such cases a multivariate one-directional test with respect to the multiple outcomes can be obtained from a combination of the individual *Z*-test values of the form
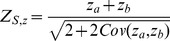
(19)where 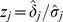 and the covariance matrix of the *Z*-tests 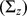 has variances 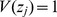 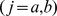 and 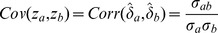 with elements from (12). If 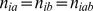 for 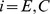 then 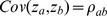.

Under the alternative hypothesis where the components 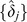 or 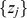 are expected to be positive, then the covariance will likewise be expected to be positive and Frick's condition 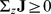 is readily satisfied. If this condition is not be satisfied, we would use the test 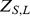 using 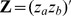 and 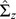 in lieu of 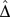 and 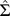 in (10).

It should be noted that this *Z*-based test is analogous to the Gastwirth [14] miximin efficient robust test (MERT) that is a obtained using the sum of the extreme *Z*-tests from a set of tests against a closed family of alternatives. For a family with only 2 alternatives (or tests), the MERT is equivalent to the above *Z*-based test.

### Comparison of the Tests for Means

When the variances are equal 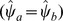, it can readily be shown that the standardized scores test equals the scale-based test 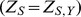 regardless of the sample sizes or sample fractions. When the group sample sizes are equal with no missing values, it can also be shown that the standardized scores test equals the *Z*-based test 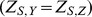. When both the variances and sample sizes are equal, then all three tests are equal.

Direct computation of the three tests 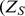, 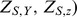 over a range of sample sizes, variances and group differences shows that 
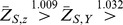
 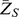, i.e. with given proportionalities. Thus, 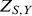 and 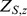 are virtually equivalent with 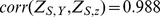 over the range of alternatives considered. These two tests are about 3% greater than the scale-based test with respective correlations of 0.977 and 0.953. Thus, on this basis the standardized scores or *Z*-based test would appear to be preferable.

### General Expressions for Power and Sample Size for the Tests

For each variation of the test, expressions for the evaluation of sample size and power are readily obtained. Under 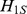 with specified values 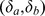, let 
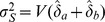
 that may be a function of 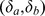 depending on the underlying model. Also, let 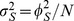 represent the factorization of this variance into a term 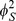 and *N*. Therefore, from standard equations [15], the power of the test to reject 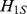 for specified values 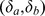 is provided by 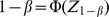 where
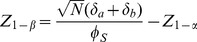
(20)and where the variance 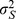 is factored as
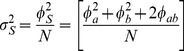
(21)and the individual variances and the covariance are factored as 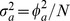, 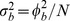, and 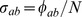. Specific expressions are presented below. Conversely, the sample size required to provide power 1−*β* to detect specified values 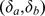 is provided by
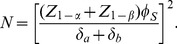
(22)


To evaluate these equations, is it necessary to provide the components of 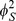, i.e. 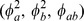, and to specify the values 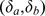 representing the minimal degree of superiority of treatment both outcomes of clinical interest.

For the standardized scores test in (16) the variance is likewise factored as 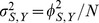. Then power is obtained from
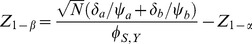
(23)and the required sample size from
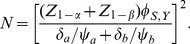
(24)


Likewise, for the *Z*-based test in (19), power is obtained from
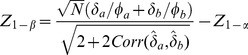
(25)and the required sample size from
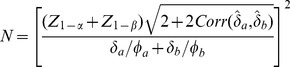
(26)where 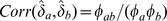. Expressions for the correlation are provided below for specific cases.

Also, each of the above expressions for power can be expressed as 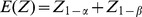 where *E*(*Z*) is also termed the non-centrality parameter of the test. Thus, the first term on the right hand side of (20), (23) and (25) is the respective expression for *E*(*Z*).

### Sample Size and Power for Tests for Means

To assess sample size and power for a test, let 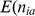 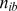 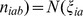 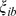 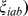 denote the expected numbers observed in the *i*th group, where *N* is the total sample size in the two groups with at least one observed measurement (not including any subject missing both *A* and *B* measurements).

### The Scale-Based Test

From (12), the covariance matrix 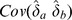 can be factored as 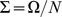 where
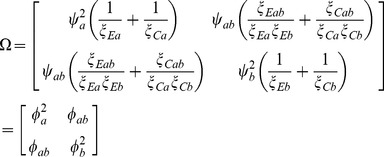
(27)and 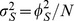 where
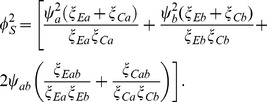
(28)


When the groups are of equal size with the same fractions observed 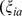 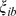 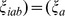 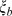 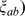 for 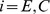, then
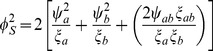
(29)


When there are equal-sized groups with no missing observations then 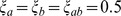 and
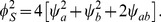
(30)


Then the power or sample size required to detect specified values 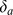 and 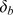 are provided by (20) or (22), respectively.

For example, suppose we desire to test the treatment group differences in both systolic (*A*) and diastolic (*B*) blood pressures, lower values of each being better. From existing data the respective SDs are 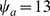 mm Hg and 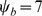 mm Hg. The correlation of the two is 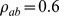 which yields 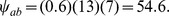 Assume that we wish to detect a treatment group difference equal to 0.25 SD for each measure, so that 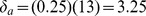
and 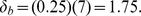 For equal-sized groups with no missing observations then 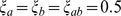 and 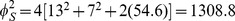. For a one-sided test at the 0.05 level, the sample size required to provide power of at least 0.9 is provided by
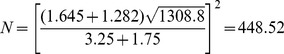
(31)or 225 subjects per group (rounded up). From equation (20), with this sample size the power to detect smaller differences of 0.2 SD with 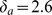 and 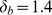, then the power using *N* = 450 is provided by
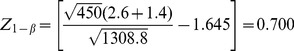
(32)with power 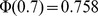. Below we also examine the power for this example using the other tests.

### The Test Using Standardized Means or Z-Scores

When the component measurements have different units or scales of measurement, then either the test based on the standardized values or the individual *Z*-tests is invariant to scale transformations, and, therefore, preferred. This test may also be preferred when the component measures have different variances, even when measured on the same scale.

For the standardized-scores test, from (16),
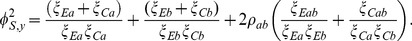
(33)


When there are equal-sized groups with no missing observations (all 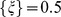) then 
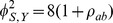
. Power and sample size are then obtained from (23) and (24).

For the above example, with equal sample sizes and no missing data, then 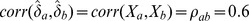. Since the difference is specified as a fraction of the standard deviation, 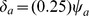
and 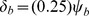, then 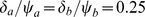 and the required sample size is
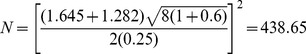
(34)that is slightly less than the *N* required for the scale-based test. This indicates that for this example, the test based on standardized scores would have greater power for a given *N*.

The same numerical result also is obtained using the *Z*-based test since in this case the two tests are equal.

### Relative Efficiency Versus Other Tests

It is also instructive to compare the efficiency of the Wei-Lachin test versus two one-sided tests or an omnibus test. We do so here in the context of a test for means, and these results apply in general to other tests as well. Standard methods for the evaluation of the asymptotic relative (Pitman) efficiency (ARE) of two tests under a local alternative would not account for the necessary adjustment to the significance level for two tests. However, the ARE can be interpreted as the ratio of sample sizes needed to provide the same level of power for a specific alternative. This ratio of sample sizes can be derived directly from (22) relative to the like expression for either two separate tests or the omnibus test.

#### Pairwise Tests

Consider the power of the test for means with equal group sample sizes and residual variance 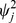 for the *j*th outcome where each is measured on the same scale so that the original scale-based test is appropriate. For a given alternative 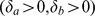. For two tests with equal-sized groups, each being of size *N*/2, with no missing data 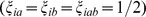, the variance of the difference for the *j*th outcome is
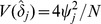
(35)assuming homoscedasticity. Then the equivalent expression for the total sample size required based on the separate tests is provided by
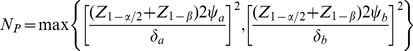
(36)using the Bonferroni correction for 2 one-sided tests. To simplify, assume that the differences of interest are a common fraction *v* of the standard deviations, i.e. 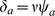 and 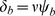 in which case
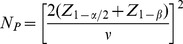
(37)


Let *N*_S_ denote the total sample size required for the Wei-Lachin test as obtained from (22) with the value 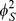 that is obtained from (30) to yield
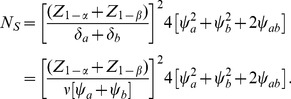
(38)


Thus, the ratio of sample sizes needed with the two-pairwise one-sided tests versus the Wei-Lachin test is
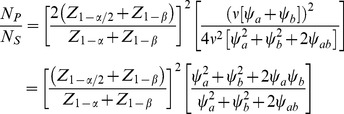
(39)


Since 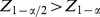 and 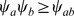, then 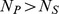.

For example, consider a one-sided test at the 0.05 level (0.025 adjusted for two tests) with 90% power to detect an improvement *E* versus *C* at any level *v*. Assume a correlation among the *A* and *B* measures of 0.5 and variances 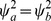  =  1. Then the ratio of sample sizes is
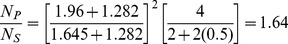
(40)which indicates that two separate tests requires a 64% greater sample size than does the Wei-Lachin test for this *α* and *β*, or that the Wei-Lachin test is 39% more efficient. These results apply approximately to other tests such as the test for proportions or the test of life-times.

#### The Omnibus MANOVA Test

Similarly, the omnibus multivariate analysis of variance (MANOVA) *T* ^2^-like test of *H*_0_ versus the general alternative *H*_1*O*_ in (7) is provided by 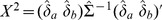 that is asymptotically distributed as chi-square on 2 *df*. The corresponding non-centrality parameter is
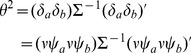
(41)where the inverse covariance matrix is
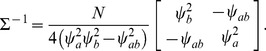
(42)


Thus
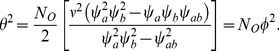
(43)


The non-centrality parameter for a test at level *α* on *K df* that provides power 1−*β*, designated as 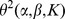, is readily obtained, such as from the SAS function CNONCT. Then the required sample size is provided by
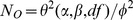
(44)


For the above example, 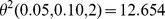 and
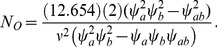
(45)


Then, for the above example, the inverse efficiency relative to the Wei-Lachin test is provided by the ratio of *N_O_* to *N_S_* in (38) to yield
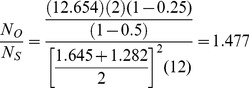
(46)and the Wei-Lachin test is 32% more efficient for these operating characteristics. If the computation is conducted for a two-sized Wei-Lachin test, then N*_O_*/*N_S_* = 1.204 and the Wei-Lachin test is 17% more efficient.

## Power of Tests for Multiple Proportions, and Mixtures of Proportions and Means

### Test for Multiple Proportions

Now consider a large sample test for a difference between groups in the probabilities 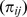 of two Bernoulli variables 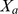 and 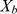 where the corresponding sample proportions are distributed as 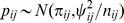 with Bernoulli variance 
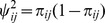
 for the *j*th outcome within the *i*th group and sample sizes 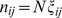, 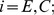 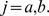 The covariance of the Bernoulli variables within the 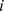
th group, 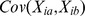, is simply
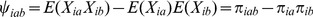
(47)where 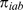 is the probability that both variables are positive [7]. Again we assume that a lower probability is better. If not, the (0, 1) categories should be reversed.

Then 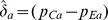 and 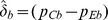 and 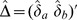 is asymptotically distributed as in (3) with expectation 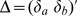 where 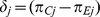 and with covariance matrix
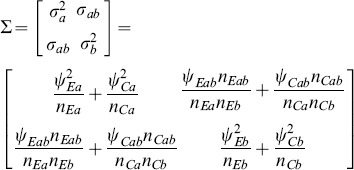
(48)that is consistently estimable from the sample quantities [7]. Then the statistic *Z_S_* is constructed as in (5) based on the sample estimate of the variance 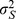 as in (13). Note that in this case, since all measures are based on Bernoulli variables, there is no advantage to using the test based on standardized scores. Alternately, the *Z*-based test would be constructed as in (19) with 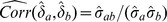.

For the assessment of sample size or power the covariance would be factored as 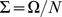 with terms 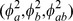 and where 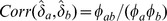.

For example, assume that the outcomes in the control group are expected to have probabilities 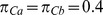 with joint probability 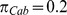 and that the respective probabilities in the experimental group are 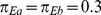 with joint probability 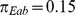. Then 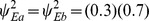, 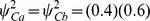, 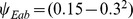, and 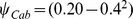. With equal sized groups and no missing observations, then 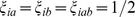 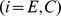 and
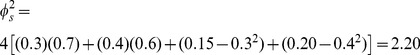
(49)with the resulting computation
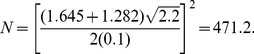
(50)


The correlation of the estimates is
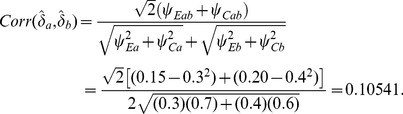
(51)


Then the test based on *Z*-test values would require
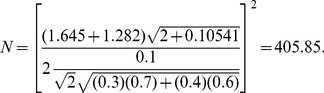
(52)


Thus, the *Z*-based test is again more efficient than the scale-based test.

### Tests for Means and Proportions

#### Scale-Based Test

It is also possible to determine the joint distribution of a test for means of one outcome and a test for proportions of another. Let 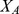 denote a quantitative measurement with means 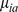 and variance 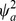, assuming homoscedasticity, and 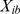 denote a binary variable with probability 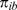 and variance 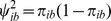 in the *i*th group 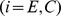. The covariance of the two in the *i*th group is provided by
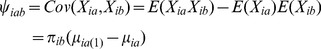
(53)where 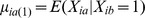 is the mean of the quantitative variable 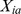 among those where the binary variable 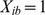. Then 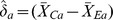 with variance 
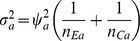
, assuming homoscedasticity, and 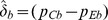 with variance 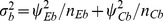. The covariance is
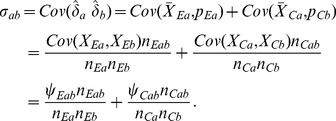
(54)


To conduct the test these variances and covariances can be estimated consistently from the corresponding sample estimates. Sample size and power can then be evaluated as above.

For example, assume that we wish to test the difference between groups in the mean level of LDL and the prevalence of hypertension. Assume a SD 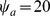 in both groups and that the difference of interest is 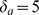 that corresponds to a 0.25 SD difference. While it is not necessary to specify the actual mean values within each group to compute 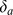, it is necessary to compute the covariance. Within each group assume that the overall mean values are 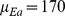 and 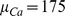 (corresponding to 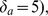 and a greater treatment effect among those who are hypertensive with mean values 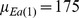 and 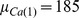. Assume that the probabilities of being hypertensive are 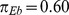 and 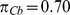, yielding 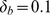. Then the variance components are 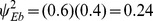, 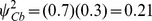, 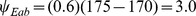, and 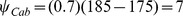.

Assuming equal sized groups with no missing data, then 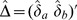 is asymptotically normally distributed with covariance matrix 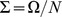 and
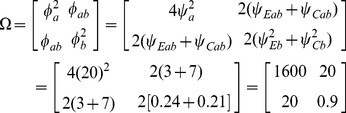
(55)and 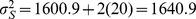. Thus, the required sample size for a one-sided test at the 0.05 level and 90% power is provided by
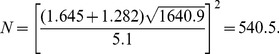
(56)


#### *Z*-Based Test

Alternately, since the scale-based test is not invariant under transformations, it would be more appropriate to employ a combination of the *Z*-tests. In this case,
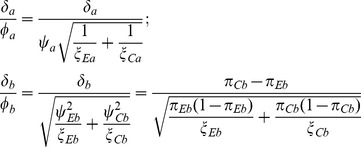


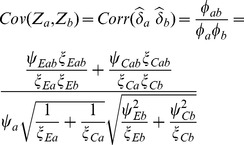
(57)


When there are equal sample sizes between groups with no missing data for either measure then
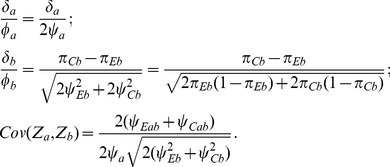
(58)


Then for this example
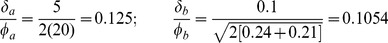
(59)

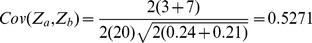
and
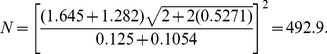
(60)


Thus, the *Z*-based test would provide greater power in this case.

## Power of Tests for Multiple Event-Times

### Tests for Multiple Event-times

For right censored event time data, a member of the family of Aalen-Gill tests [16], [17], also known as the 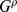 family of tests of Harrington and Fleming [18], can be used to test the hypothesis of equal hazard functions, or survival functions, between two groups. This family includes the logrank test that is asymptotically fully efficient under a proportional hazards model and is equivalent to the score test of the unadjusted group effect in a Cox Proportional Hazards model. It also includes the Peto-Peto-Prentice modified Wilcoxon test that is optimal under a survival proportional odds model. Andersen, Borgan, Gill and Keiding [19] describe a generalization of the tests for *K*>2 groups. These tests are equivalent to the family of weighted Mantel-Haenszel statistics described by Kalbfleisch and Prentice [20].

Wei and Lachin [2] describe a multivariate rank test for event times that is a generalization of the above families of tests to the case of multiple time-to-event outcomes. They also introduced the one-directional multivariate test described herein, what they termed the test of stochastic ordering, to assess whether the treatment group event times differed in a favorable direction for all of the outcomes. A SAS macro for these computations is available (see discussion). The computational details will not be provided herein.

Lakatos [21] presents a general approach to the evaluation of sample size and power for the Mantel-logrank test that allows for time varying hazard rates, proportional or non-proportional hazards, and other design features. When the hazard rates are assumed constant over time with a constant of proportionality, a simple exponential model applies in which case the methods of Rubenstein et al. [22] or Lachin and Foulkes [23] can be applied. Herein we describe the computation of sample size or power for the Wei-Lachin test for multiple event-time outcomes under the exponential model of Lachin and Foulkes that includes a generalization of the method described by Lachin [15] based on the difference in the exponential hazard rates. Freedman [24] showed that the latter expression can also be derived from the expected value of the logrank chi-square test value under a proportional hazards model. Lachin and Foulkes [23] also show that the power of the test based on the difference in the estimated hazards is virtually identical to that for a test based on the log hazard ratio.

We assume that there are two or more outcome events where no one outcome is a competing risk for the other outcomes, such as the time to development of diabetic retinopathy and time to developing diabetic nephropathy, neither of which is fatal. Let *X_ijk_* = 1 denote that the *k*th subject had the *j*th event in the *i*th group at time *t_ijk_*, and *X_ijk_* = 0 denote right censoring at time *U_ijk_* that in turn is the minimum of the loss to follow-up time and the administrative censoring time for those who remain free of the *j*th outcome, *i* = *E*, *C*; *j* = *a*, *b*. Then the total number of subjects with an event (called events) (*D_ij_*) and total time at risk (*T_ij_*) for the *i*th group and the *j*th outcome are
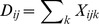
(61)

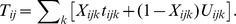



Note that the *X_ijk_* are non-iid Bernoulli variables with event probabilities that are a function of the underlying hazard rates for the event and losses to follow-up and the period of exposure *U_ijk_*.

Within each group, for each outcome assume a constant hazard rate 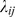 that is consistently estimated as 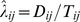. Let 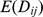 designate the expected number of events based on the assumed hazard rate 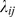, sample size, periods of recruitment and follow-up, and losses-to follow-up in that group. Asymptotically,
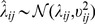
(62)where 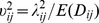 that is consistently estimated as 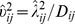.

Then 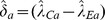, 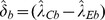 and, 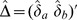 is asymptotically distributed as in (3) with expectations 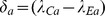 and 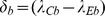 and covariance matrix 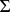 with elements 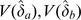 and 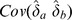. A test based on 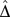 will have power approximately equal to that of the Wei-Lachin multivariate one-directional test using the Wei-Lachin bivariate Aalen-Gill logrank test under proportional hazards. Thus, we describe the power of the bivariate logrank test based on the test of the difference in exponential hazards. Then the scale-based test employs
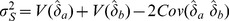
(63)

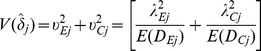
that is consistently estimated using 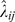 and the observed 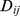, 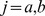.

File S1 shows that the covariance is expressed as
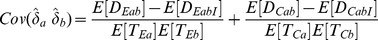
(64)where 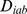 is the number of subjects who experience both the 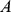 and 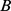 events and 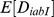 is the expected number with both events under the assumption that the Bernoulli variables 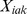 and 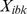 are independent. Each is consistently estimated from the observed numbers of events and total time at risk. The computational expression for 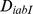 is also presented in File S1.

The resulting test as in (5) then is based on the variance estimate
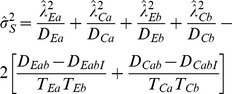
(65)that is solely a function of the numbers of individual and joint events, the corresponding event times and the corresponding times of at risk. Accordingly, the power of the test is a function of the expected numbers of events and expected time at risk that in turn are a function of the design parameters and sample size.

Lachin and Foulkes [23] provide the expression for the probabilities of events 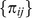 for given hazard rates for events 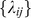 and losses to follow-up 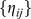, recruitment period 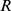 with recruitment shape parameter 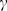 and total follow-up *Q*, and sample size 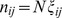. Then the expected number of events is obtained as 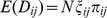 and likewise the expected period at risk as 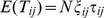. File S1 also provides expressions for 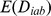, 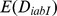 and 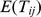. Then 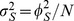 where
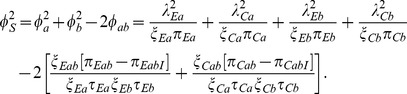
(66)


Power and sample size are then obtained from (20) and (22).

However, to obtain an analytic solution to these equations, a specific model must be specified for the dependence of the event-times with a given correlation, such as the Marshall and Olkin [25] bivariate exponential model. Hougaard [26] provides a review of such models. Alternately, a simulation model could be implemented using a given bivariate exponential distribution. Herein, a simpler approach is described using a shared frailty.

Assume that the two event types share a common frailty with parameter 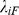. Then in the simulation model, in the *i*th group, three random exponential times are generated as
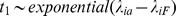


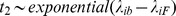


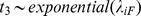
and the correlated exponential event times are then obtained as
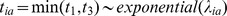


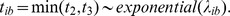



from which the probability 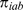 of both events can be obtained.

For example, consider a 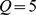 year study with linear (constant) recruitment over a 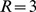 year interval allowing for a loss-to-follow-up hazard rate of 0.05 per year and with equal size groups. Within the control group assume that the hazard rates are 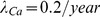 and 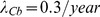 and that the experimental therapy yields risk reductions of 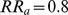 and 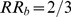, or hazard rates of 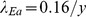 and 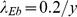 so that 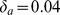 and 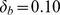. To allow for a correlation of the event times we assume shared frailties of 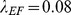 and 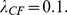 For a given sample size, the simulation model (herein with 10,000 replications) provides direct computation (within a small degree of error) of the expected quantities (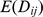, etc.) from which power is computed. By a simple search it was found that a *n* of 197 per group provides a one-sided one-directional test with 90% power.

For this sample size, the expected number of events marginally are 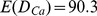, 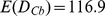, 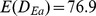, and 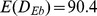; and the expected patient-years at risk are 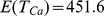, 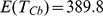, 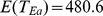, and 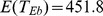. The numbers of subjects with both events with the shared frailty are 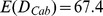 and 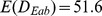, and those expected under independence (by chance) are 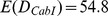 and 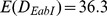. These yield 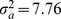
E–4, 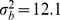
E–4, and 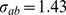
E–4, that provides 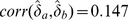 and 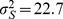
E–4. Substituting into (20), yields 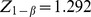 and power  =  0.902.

A similar computation using (25) shows that an *n* of 197 per group would provide power  =  0.885 using the *Z*-based test, indicating that in this setting the *Z*-based test would have less power than the original scale based test.

### Generalizations

It is also possible to obtain a test based on the combination of group differences in hazard rates and differences in proportions or means. As in the preceding sections this requires the derivation of the covariance of the measures within each treatment group.

Alternately, a multivariate one-directional test can be obtained using multiple regression models as now described.

## Model-Based Analysis of Multiple Outcomes

The preceding sections describe the application of the Wei-Lachin test to a combination of the group differences in means or proportions or hazard rates. In each case the covariance of the group differences, or of the corresponding *Z*-values, is described. The test statistic can then be computed using a consistent sample estimate of the variances and covariance(s), and the expression for power can be obtained using specified values for these parameters. In principle it is possible to construct a test for combinations of other types of outcomes, such as the difference in rates (counts) of events under a Poisson model, and to derive the equations to assess the power of the tests. However, it is more convenient to provide model-based generalizations of this approach.

From basic principles, Pipper, Ritz and Bisgaard [27] describe the joint distribution of parameter estimates from multiple models, not necessarily all of the same type. Consider two models for each of two outcomes, each with *K_j_* parameters and coefficient estimates 
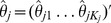
. Arbitrarily, assume that the first parameter estimate 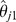 represents the difference between groups on some scale, no difference represented by a value of zero, and the remaining *K_j_* estimates represent the intercept (if any) and other covariate effects. Then 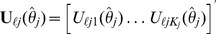 is the score vector for the 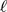
th subject and 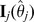 is the model based estimate of the expected information for the *j*th outcome. Also, let 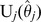 denote the 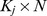 matrix where the 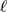
th column is the score vector 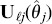. Then the generalization of the information sandwich robust estimate of the covariance matrix of the joint set of estimates 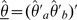 is provided by
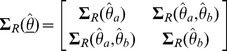
(67)where
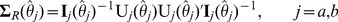
(68)

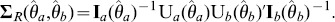



The estimated variances of the group coefficients in the two models is then provided by the elements 
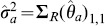
 and 
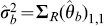
, and the covariance by 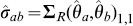. The scale-based test is then provided by (5) with 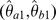 substituted for 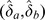. Alternately, *Z*-tests of the group effect in the two models are then provided by 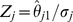, 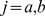, and the correlation of these tests by 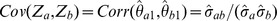. This provides the *Z*-based test as in (19).

Pipper et al. also describe the application of the joint models where data for a subject is missing for one of the component models (but not both). Under the assumption of missing completely at random, then the score vector elements for that subject are set to zero in the corresponding score matrix U.

It would be difficult to evaluate the sample size and power of such a model-based test. However, simple computations such as those herein could be applied, e.g. the power of a test for a difference in means and proportions when the actual analysis will employ a linear regression model and a logistic model.

Pipper et al. originally provided an *R* package *multmod* to fit multiple models and to compute the covariances of the coefficients in the models. That has since been replaced by the *R* package *multcomp*.

## Example – The Diabetes Prevention Program

The Diabetes Prevention Program compared the risk of onset of type 2 diabetes and deterioration of metabolic function among participants randomly assigned to an intensive lifestyle intervention (ILS) versus treatment with the glucose lowering drug metformin and versus a placebo control with no lifestyle intervention [28]. The study showed that intensive lifestyle provided a 58% reduction in diabetes risk versus placebo and 39% versus metformin, and that metformin produced a 31% reduction versus placebo. The study also evaluated the differences among treatments in the prevalence of developing the *metabolic syndrome*, a metabolic state that is linked not only with risk of onset of diabetes but also the risk of developing cardiovascular disease. The prevalence of the metabolic syndrome is characterized by 3 or more of the following 5 criteria: abdominal obesity defined as a waist circumference >102 cm among men or >88 cm among women, serum triglycerides (a bad cholesterol) ≥150 mg/dL, HDL (a good cholesterol) <40 mg/dL among men or <50 mg/dL among women, systolic/diastolic blood pressure ≥130/85 mm Hg, and fasting glucose ≥110 mg/dL, the latter met by many of the study subjects. [29]

Of the 3234 randomized, 1388 (43%) already met the metabolic syndrome criteria. Among the remainder who were evaluated at 3 years of follow-up (i.e., free of the syndrome on entry), 22% (363 of 1673) had the syndrome present. [30] Herein we compare the prevalence of the metabolic syndrome and its components at 3 years of follow-up among those in the lifestyle versus metformin treated groups.

The classification of the metabolic syndrome is a composite outcome, i.e. a single binary trait to designate that the criteria were met. An alternative would be to construct an analysis of the 5 binary traits using the one-directional multivariate test described herein.

For two of the traits (waist circumference and HDL) there are separate criteria for men and women, and for hypertension both systolic and diastolic blood pressure are employed, whereas for the other two traits there is a single cutpoint for the corresponding quantitative measure. Thus an alternate analysis would be to used these three composite binary traits in conjunction with an analysis of the other two quantitative variables (triglycerides and glucose). Alternately, rather than use any cutpoints to construct derived binary variables, an analysis could compare the groups with respect to the six quantitative traits (including systolic and diastolic blood pressure) simultaneously.

Table 1 presents a comparison of the lifestyle versus metformin groups for each of the binary outcomes and each of the corresponding quantitative outcomes. The overall prevalence of the metabolic syndrome using the composite binary outcome does not differ significantly between groups, although the prevalence is about 2% lower in the lifestyle group.

**Table 1:** Differences between the DPP intensive lifestyle (ILS, *n* = 571) versus metformin (MET, *n* = 557) treated patients at three years of follow-up with respect to quantitative trait components of the metabolic syndrome, and binary indicators of abnormal levels, and the overall incidence of the metabolic syndrome among those free of the syndrome on entry.

	Mean (SE)			%		
Characteristic	ILS	MET	*p*	ILS	MET	*p*
Waist (cm)	97 (0.61)	99 (0.60)	0.0030	54.6	63.4	0.0015
Triglycerides (mg/dl)	115 (2.5)	125 (2.9)	0.0017	19.3	25.3	0.0074
HDL (mg/dl)	51.3 (0.53)	50.7 (0.53)	0.10	36.6	37.9	0.33
BP hypertension				9.5	9.3	0.53
Systolic (mm Hg)	120 (0.64)	122 (0.60)	0.0046			
Diastolic (mm Hg)	74 (0.40)	76 (0.37)	0.0001			
Glucose (mg/dL)	104 (0.49)	103 (0.53)	0.59	24.1	23.5	0.60
Metabolic Syndrome				18.2	20.1	0.22

Analysis restricted to those free of the metabolic syndrome at entry. One-sided p-values computed from a t-test for quantitative measures and chi-square test for binary variables.

For all variables other than HDL, higher values are worse, so that a positive difference between metformin minus lifestyle indicates a benefit for lifestyle. In order for the same to apply to HDL, the analysis employed the negative values of HDL.

All *p*-values are one-sided. Some of the one-sided *p*-values are >0.5 indicating a negative *Z*-value favoring metformin. However, most of these differences are close to zero. For no measure is there evidence that intensive lifestyle is worse than metformin, and all significant differences favor the lifestyle group. Thus, these data are consistent with the alternative hypothesis that lifestyle has a beneficial effect on some of the outcomes, and no adverse effect for any.

Table 2 presents the correlations among the measurements. The modest to low correlations suggest that a multivariate test will provide greater power than individual tests, especially when the latter are adjusted for multiple tests.

**Table 2:** Correlations among the component measurements obtained from the pooled within-groups covariance matrix.

	Triglycerides	HDL	SBP	DBP	Glucose
Waist (cm)	0.07	0.24	0.13	0.19	0.28
Triglycerides (mg/dl)		0.27	0.03	0.11	0.06
HDL (mg/dl)			−0.09	0.04	0.14
Systolic (mm Hg)				0.55	0.08
Diastolic (mm Hg)					0.05

Table 3 then presents the Wei-Lachin scale-based and Z-based one-directional multivariate test Z and one-sided p-values for three different analyses of these data. As would be expected, the analysis of all six quantitative traits is more powerful or sensitive than the analyses involving binary traits, with *p*-values <0.001 using either the scale or *Z*-based tests. The analysis of the 5 binary indicator variables produces less significant results, and the scale-based test for these data proves to be more powerful (larger *Z*-value) than the *Z*-based test, although both are significant. An alternative would be to conduct an analysis of the three binary traits defined from multiple criteria (waist, HDL, hypertension) and the other two quantitative traits (triglycerides and glucose). This yields results intermediate to those of the analysis of all quantitative and all binary traits.

**Table 3:** The Wei-Lachin scale-based and Z-based one-directional multivariate test Z and one-sided p-values for three different analyses of the DPP metabolic syndrome data.

	Scale-based Test		*Z*-based Test	
Analysis	*Z_s_*	*p_s_*	*Z_S,z_*	*p_S,z_*
All quantitative (6)	3.52	0.00022	3.48	0.00025
All binary (5)	2.37	0.0089	2.22	0.0131
Mixed (5)	2.49	0.0064	2.02	0.0215

Regardless of which of these options might have been chosen as the basis for the analysis, all would have provided a statistically significant result whereas the analysis of the composite metabolic syndrome outcome failed to demonstrate a beneficial effect of lifestyle versus metformin (Table 1, *p* = 0.22).

## Discussion

A number of multivariate one-directional or one-sided tests have been described. Virtually all were developed to apply to a multivariate test of the difference in means between two groups for a multivariate outcome, such as repeated measures. These are also described for the case of two measures with group differences 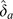 and 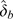 as described above.

For a test based on multivariate normal observations, such as *K* repeated measures, Kudo [31] described the multivariate one-sided likelihood ratio test (*LRT*) of the *K*-variate generalization of the ordered hypotheses in (2) assuming that the covariance matrix 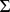 is known, and Pearlman [32] described the *LRT* when the estimated covariance matrix is employed. For the case of the two statistics herein, Pearlman's *LRT* is based on the statistic
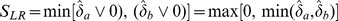
(69)where “
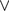
” designates the maximum of the two quantities. Thus, if either 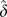 is negative the resulting test statistic quantity is zero. However, the distribution of *S_LR_* is computationally difficult and the test is not convenient for practical use.

Tang, Gnecco and Geller [33] proposed a computationally simpler approximation to the *LRT*. Their approximate or *ALR* test is not an approximation in the sense, say, of a series expansion, but rather is an approximation in the sense that the alternative hypothesis parameter space is an approximation of that of the *LRT*. Their statistic is of the form
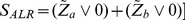
(70)where 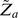 and 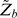 are uncorrelated standardized *Z*-statistics obtained as linear transformations of the 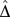 vector. Under the assumption that the covariance matrix is known, then 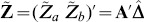 where **A** is a square matrix such that 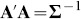 and 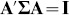, such as is obtained from a Choleski decomposition. The distribution of this statistic is a simplified Chi-bar-squared distribution [34], though still requiring some computation to obtain a *p*-value. However, when an estimate of the covariance matrix is employed to provide the **A** transformation matrix, various authors have shown that the test can be serverely liberal, i.e. has an inflated type I error probability. In this case, Tamhane and Logan [35] described an accurate approximation to the distribution of the resulting test using a mixture of *F*-distributions, that also requires some computation to determine levels of significance.

However, this test has the unsavory feature that if either 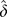 value is negative, regardless how greatly so, the value is set to zero in the computation of the test statistic. Thus, for example if 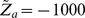 and 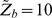, then 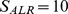, and depending on the estimated covariance values, could reject *H*_0S_ in favor of *H*_1S_, even though it is clear that *H*_1S_ does not apply. In a recent overview, Tamhane and Logan [36] have suggested that “If several endpoints show moderate negative differences or even if a few show very large negative differences, then these tests should not be used because the *a priori* assumption of positive treatment effects in all endpoints is questionable.” However, to apply this recommendation in practice violates the principle that the test statistic for a study be specified *a priori*. In effect, the recommended practice could be viewed as a two-stage inference process - first determine if the differences are positive, and if so conduct the test. This would clearly inflate the type I error probability.

Other tests have been proposed that are based in part on Hotelling's *T* ^2^ statistic that is equivalent to the expression in (8) and is distributed as *T* ^2^ on *K df* under the assumption of multivariate normality of the observations. Under this assumption, *T* ^2^ provides an optimal test of the null hypothesis against the global alternative presented in (7). Follman [37] describes a test of *H*_0_ versus *H*_1+_: 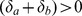 that is not the same as *H*_1S_ above. His 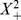 test rejects *H*_0_ in favor of *H*_1+_ if *T* ^2^ is significant at level 2*α* and 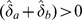. This test also could lead to rejection of *H*_0_ when either the true 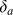 or 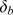 is a large negative value and the other an even larger positive value.

Bloch, Lai and Tubert-Bitter [38] describe another test procedure which requires that *T* ^2^ reach significance at level *α* two-sided and that both individual one-sided *t*-tests of an indifference hypothesis be significant at level *α*. The indifference hypothesis is *H*_0*I*_: 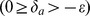 and 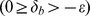 for some small positive value 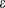, and the alternative hypothesis is *H*_1S_ as in (2) above so that the one-sided *t*-test is of the form
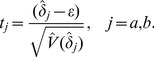
(71)


This test was later criticized by Pearlman and Wu [39] who proposed use of the one-sided *LRT* of Pearlman [32] in lieu of *T* ^2^, among other improvements. The result of either test, however, depends on the specification of the value 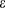 and thus the test may not be uniformly acceptable.

Other tests have also been applied, although not specifically designed to test *H*_0_ against the one-sided alternative *H*_1S_ in (2). O'Brien [13] proposed his ordinary least squares (*OLS*) and weigthed least squares 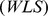 tests of *H*_0_ versus the alternative hypothesis of a common difference *H*_1*A*_: 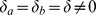. Thus the alternative hypothesis consists of the line of equality other than the origin. The one-sided version of this test will also be sensitive to alternatives where 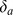 and 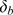 are of similar positive magnitude, but will not be optimal against the general alternative *H*_1S_. Pocock, Geller and Tsiatis [40] describe the application of these tests to the analysis of multiple outcomes in clinical trials on different scales.

For a two group comparison of a vector of repeated measures, under the usual normal errors assumptions O'Brien also suggested that his statistics were distributed as *t*. However, the exact small sample distribution with normal errors is not known and many authors have shown that the resulting *t*-statistics have an inflated type I error probability. For a vector of repeated measures in two groups, Läuter [41] shows that statistics that employ weighted averages, as in O'Brien's *WLS* test, are indeed distributed as *t* provided that the weights are functions of the empirical covariance matrix estimated from all groups combined rather than the pooled within-groups covariance matrix estimate as employed by O'Brien. He proposes a family of such weighted tests that includes the Wei-Lachin test as a trivial special case. Frick [42] also showed that O'Brien's OLS test is biased.

Thus, among the various tests that have been proposed that could be applied to the assessment of simultaneous differences between groups for multiple outcomes, the Wei-Lachin test has the advantages that it is simple to compute; can be applied to mixtures of outcomes on different scales (e.g. means and proportions); that it has a large sample normal distribution (or a *t*-distribution with normal errors); provides a test with type I error probabilities close to the nominal levels with generally acceptable sample sizes; is directed towards the specific multivariate one-directional alternative of interest, is maximin efficient relative to the possible true but unknowable optimal test, and readily provides for the computation of sample size and power.

Rahlfsand Vester [43] describe applications of the Wei-Lachin test to the analysis of multiple outcomes using the multivariate Mann-Whitney difference analysis described initially by Thall and Lachin [11]. The authors are affiliated with idv Data Analysis and Study Planning that also markets a program (TESTIMATE) that conducts such Wei-Lachin analyses. Pan [44] also recently presented a review of various procedures including the Wei-Lachin test (called the SUM test therein) and some of the above referenced one-directional procedures and showed by simulation that the Wei-Lachin test had good power when the outcomes tended to jointly show beneficial effects.

Programs for computations herein are available from www.bsc.gwu.edu. These include the coefficient vector *L* for use in (10) when Frick's condition does not apply, the simulation event time model, and the Wei-Lachin multivariate rank test.

### Ethical Statement

Neither animals or human subjects were involved in this methodological research.

## Supporting Information

File S1 (PDF)
