# Supplementary material for: Applications of the Wei-Lachin Multivariate One-Sided Test for Multiple Outcomes on Possibly Different Scales
Source: PLoS One. 2014 Oct 17;9(10):e108784. doi: 10.1371/journal.pone.0108784 (PMC4201485; doi:10.1371/journal.pone.0108784)
Supplement: File S1 — (PDF) [file pone.0108784.s001.pdf]

Applications of the Wei-Lachin Multivariate One-Sided Test for Multiple Outcomes on Possibly Different Scales

John M. Lachin[1,*]
1 The Biostatistics Center, The George Washington University, Rockville, MD, USA
$*$ **E-mail: jml@bsc.gwu.edu**

Supporting Information

A Expected Event Probabilities and Durations at Risk.

Let λ_{ij} denote the hazard rate for the jth event in the ith group with sample size $N\xi_i$, assuming that each subject is observed for some fraction of time for each event type, i.e. that $\xi_{ia} = \xi_{ib} = \xi_{iab} = \xi_i$. Herein we assume that all subjects are at risk for some time for all events in which case the same sample fractions apply within the ith group. Then let π_{ij} denote the probability that a subject in the ith group reaches the jth event marginally, i.e. either alone or jointly. Lachin and Foulkes (1986) assume recruitment over an interval of $(0, R]$ according to a monotone increasing function $G(r) = \int_0^r g(u)du$. For uniform (linear) recruitment, $g(r) = 1/R$. For non-linear recruitment, Lachin and Foulkes use a truncated exponential distribution with density

$$g(r) = \frac{\gamma e^{-\gamma r}}{1 - e^{-\gamma R}}, \tag{1}$$

for $0 < r \le R$, and $\gamma \ne 0$, that yields a concave (delayed) recruitment pattern for $\gamma < 0$, or a convex (accellerated) pattern for $\gamma > 0$. The total study duration is $Q > R$ units of time so that the administrative censoring time for a subject is then $Q - r$. Lachin-Foulkes also assume exponential losses with hazard rate η_{ij} assuming that the loss times for the two events are independent, with $h_{ij}(u) = \eta_{ij} e^{-\eta_{ij} u}$ and cumulative distribution function $H_{ij}(u) = 1 - e^{-\eta_{ij} u}$. Then the expected potential exposure time (not factoring events) is

$$E(U_{ij}) = \int_{r=0}^{R} g(r) \left(\int_{u=0}^{Q-r} u \; h_{ij}(u) \; du + (Q-r) \left[1 - H_{ij}(Q-r)\right] \right) dr \tag{2}$$

that is readily evaluated using numerical integration. Note that in this instance we allow for different risks of loss-to-follow-up for the different types of events within and between groups. Then the probability of the ijth event (π_{ij}) with linear or non-linear exponential recruitment is provided by equations (4.1) or (4.3), respectively, in Lachin-Foulkes. Note that these equations are of the form $\lambda^2\{\pi\}^{-1}$, so that the term in braces is the quantity of interest. Then

$$E(D_{ij}) = N\xi_{ij}\pi_{ij} \tag{3}$$

The expected time at risk for the ijth event, i.e. time to event or right censoring, is provided by

$$\tau_{ij} = \int_{r=0}^{R} g(r) \left(\begin{array}{c} \int_{x=0}^{Q-r} x \; f_{ij}(x) \; [1 - H_{ij}(x)] \; dx \\ + \int_{u=0}^{Q-r} \; u \; h_{ij}(u) \; [1 - F_{ij}(u)] \; du \\ +(Q-r)\left[1 - H_{ij}(Q-r)\right]\left[1 - F_{ij}(Q-r)\right] \end{array} \right) dr \tag{4}$$

that is readily evaluated by numerical integration. Then

$$E(T_{ij}) = N\xi_i\tau_{ij} \tag{5}$$

that is the expected denominator for the ijth hazard rate estimate.

B Covariance of Exponential Hazard Differences.

Consider the case of two exponentially-distributed event times, neither being a competing risk for the other, with group differences $\widehat{\delta}_a = (\widehat{\lambda}_{Ca} - \widehat{\lambda}_{Ea})$ and $\widehat{\delta}_b = (\widehat{\lambda}_{Cb} - \widehat{\lambda}_{Eb})$. To obtain the covariance $Cov(\widehat{\delta}_a \; \widehat{\delta}_b)$, it is readily shown that

$$\begin{aligned} Cov(\widehat{\delta}_a \; \widehat{\delta}_b) &= Cov\left[\frac{D_{Ea}}{T_{Ea}} - \frac{D_{Ca}}{T_{Ca}}, \frac{D_{Eb}}{T_{Eb}} - \frac{D_{Cb}}{T_{Cb}}\right] \\ &= Cov\left[\frac{D_{Ea}}{T_{Ea}}, \frac{D_{Eb}}{T_{Eb}}\right] + Cov\left[\frac{D_{Ca}}{T_{Ca}}, \frac{D_{Cb}}{T_{Cb}}\right] \\ &= \frac{Cov(D_{Ea}, D_{Eb})}{T_{Ea}T_{Eb}} + \frac{Cov(D_{Ca}, D_{Cb})}{T_{Ca}T_{Cb}}. \end{aligned} \tag{6}$$

Within the ith group,

$$\begin{aligned} Cov\left[D_{ia}, D_{ib}\right] &= E\left[D_{ia}D_{ib}\right] - E\left[D_{ia}\right]E\left[D_{ib}\right] \\ &= E\left[\left(\sum\nolimits_k X_{iak}\right)\left(\sum\nolimits_k X_{ibk}\right)\right] - E\left[\sum\nolimits_k X_{iak}\right]E\left[\sum\nolimits_k X_{ibk}\right] \\ &= \sum\nolimits_k Cov\left[X_{iak}X_{ibk}\right] = \sum\nolimits_k \left(E\left[X_{iak}X_{ibk}\right] - E\left[X_{iak}\right]E[X_{ibk}]\right) \\ &= \sum\nolimits_k \left(\pi_{iabk} - \pi_{iak}\pi_{iak}\right) = E\left[D_{iab}\right] - E\left[D_{iabI}\right] \end{aligned} \tag{7}$$

where π_{iabk} is the probability of the kth subject experiencing both events and π_{ijk} is the probability of experiencing the jth event, both a function of the entry time of the kth patient and the other design parameters. Thus, D_{iab} is the number of subjects who experience both the A and B events and $E\left[D_{iabI}\right]$ is the expected number of subjects with both events under the assumption that the Bernoulli variables X_{iak} and X_{ibk} are conditionally independent.

Thus,

$$Cov(\widehat{\delta}_a \; \widehat{\delta}_b) = \frac{E\left[D_{Eab}\right] - E\left[D_{EabI}\right]}{E\left[T_{Ea}\right]E\left[T_{Eb}\right]} + \frac{E\left[D_{Cab}\right] - E\left[D_{CabI}\right]}{E\left[T_{Ca}\right]E\left[T_{Cb}\right]} \tag{8}$$

that is consistently estimated from the observed numbers of events and total time at risk. The sample estimate D_{iabI} is computed as

$$D_{iabI} = \sum\nolimits_k \widehat{E}[X_{iak}]\widehat{E}[X_{ibk}] = \sum\nolimits_k \left[1 - \exp(-\widehat{\lambda}_{ia}U_{iak})\right]\left[1 - \exp(-\widehat{\lambda}_{ib}U_{ibk})\right]. \tag{9}$$

For the evaluation of sample size or power, these quantities are easily obtained from a simulation model.

Alternately, from a model that provides hazard rates for the individual and joint events under the study design, the expected event counts can be obtained as

$$\begin{aligned} E\left[D_{iab}\right] &= \pi_{iab}N\xi_i \\ E\left[D_{iabI}\right] &= \pi_{iabI}N\xi_i \end{aligned} \tag{10}$$

where π_{iab} is obtained from the Lachin-Foulkes equations cited above using the hazard rate for experiencing both events λ_{iab}. The probability of experiencing both events assuming independence can then be obtained from

$$\pi_{iabI} = \int_{r=0}^{R} g(r) \;\; \left(\left[1 - \exp(-\lambda_{ia}U_{ia}(r))\right]\left[1 - \exp(-\lambda_{ib}U_{ib}(r))\right]\right) \; dr \tag{11}$$

where the expected exposure for a subject with recruitment at time r is obtained from (2) as

$$U_{ij}(r) = \int_{u=0}^{Q-r} u \; h_{ij}(u) \; du + (Q - r)\left[1 - H_{ij}(Q - r)\right] \tag{12}$$
